# Role of Neuromuscular Blocking Agents in Acute Respiratory Distress Syndrome: An Updated Meta-Analysis of Randomized Controlled Trials

**DOI:** 10.3389/fphar.2019.01637

**Published:** 2020-01-29

**Authors:** Xue-biao Wei, Zhong-hua Wang, Xiao-long Liao, Wei-xin Guo, Tie-he Qin, Shou-hong Wang

**Affiliations:** Department of Gerontological Critical Care Medicine, Guangdong Provincial Geriatrics Institute, Guangdong Provincial People's Hospital, Guangdong Academy of Medical Sciences, Guangzhou, China

**Keywords:** neuromuscular blocking agents, acute respiratory distress syndrome, meta-analysis, mortality, barotrauma, weakness

## Abstract

**Background:**

The therapeutic role of neuromuscular blocking agents (NMBA) in patients with acute respiratory distress syndrome (ARDS) remains controversial.

**Methods:**

We systematically reviewed randomized controlled trials investigating the use of NMBA in ARDS patients from inception to July 2019. Relative risk (RR) was calculated for the incidence of barotrauma and mortality using the random-effect or fixed-effect model according to heterogeneity analysis.

**Results:**

Data were combined from five randomized controlled trials that included 1,461 patients (724 in the NMBA group and 737 in the control group). Pooled analysis showed that NMBA infusion did not reduce 28-day mortality (RR = 0.72, 95% confidence interval (CI) 0.44 to 1.17, *P*=0.180, I-squared = 62.8%), but was associated with lower intensive care unit (ICU) mortality (RR = 0.60, 95% CI 0.41 to 0.88, *P* = 0.009, I-squared = 9.2%). In addition, the incidence of barotrauma was significantly lower in patients treated with NMBA (RR = 0.53, 95% CI 0.33 to 0.84, *P* = 0.007, I-squared = 0). However, infusion of NMBA might increase the risk of ICU-acquired weakness (RR = 1.34, 95% CI 0.97 to 1.84, *P* = 0.066, I-squared = 0).

**Conclusion:**

Infusion of NMBA could reduce ICU mortality and the incidence of barotrauma. The risk of ICU-acquired weakness was higher in moderate-to-severe ARDS patients treated with NMBA. The real effects of NMBA need to be further evaluated and confirmed by a study with a stricter design.

## Introduction

Acute respiratory distress syndrome (ARDS) is a life-threatening lung condition characterized by refractory hypoxemia and decreased lung compliance (Fan et al., 2018; Papazian et al., 2019). In spite of advanced therapeutic techniques, ARDS is still associated with poor prognosis. Epidemiological data have revealed that hospital mortality is approximately 35–46%, depending on the severity of ARDS (Bellani et al., 2016). Therefore, identification of an effective treatment method is of utmost importance for ARDS patients.

The primary reason for hypoxemia and hypercarbia in ARDS is an increased shunt fraction due to ventilation/perfusion mismatch (Sweeney and McAuley, 2016). These gas exchange abnormalities lead to increased minute ventilation and patient-ventilator dyssynchrony. A previous study indicated that patients with greater numbers of dyssynchronies have poorer prognosis (Blanch et al., 2015). Neuromuscular blocking agents (NMBA) have been recommended for ARDS patients with a PaO_2_/FiO_2_ ratio <150 mmHg, especially for mechanically ventilated patients with ventilator dyssynchrony (Murray et al., 2016). A recent meta-analysis indicated that a 48 h NMBA infusion might reduce intensive care unit (ICU) mortality in patients with moderate-to-severe ARDS (Tao et al., 2018). In addition, NMBA has been associated with a lower risk of barotrauma, while it did not seem to increase the risk of ICU-acquired weakness (Neto et al., 2012; Alhazzani et al., 2013). However, the benefit of NMBA was not confirmed by a recently published randomized controlled trial, the Reevaluation of Systemic Early Neuromuscular Blockade (ROSE) trial (Moss et al., 2019), leaving the use of NMBA in ARDS patients unclear and controversial. Accordingly, this updated meta-analysis includes the ROSE trial to further assess the effect of NMBA on mortality, and better understand its use and adverse outcomes in the literature published to date.

## Materials and Methods

### Search Strategy

PubMed, Web of Science, ClinicalTrials.gov, and the Cochrane Library were searched to find randomized controlled trials that evaluated the use of NMBA in ARDS patients from inception to July 2019. Keywords used to perform the search were “neuromuscular blockade” or “neuromuscular blocking agent” or “neuromuscular blocker” or “cisatracurium” and “acute respiratory distress syndrome” or “ARDS” or “acute lung injury”. We also reviewed the potentially eligible studies from the references of the previous published meta-analysis. The language was limited to English.

### Selection Criteria and Endpoints

The search results were independently scanned by two of the authors (WG and XL). Any disagreements were resolved by consensus with a third author (SW). Randomized controlled trials comparing infusion of NMBA vs. non-NMBA in ARDS patients were included. Exclusion criteria were as follows: (Papazian et al., 2019) duplicate publications; (Fan et al., 2018) non-randomized controlled trials; and (Bellani et al., 2016) lack of information on pre-defined endpoints. The endpoints included 28-day and ICU mortality, barotrauma, and ICU-acquired weakness.

### Data Extraction and Quality Assessment

The following data were extracted from all included studies: first author's name, publication year, definition of ARDS, patients' characteristics, sample size, treatment protocol, and outcomes. Quality was assessed by the Cochrane Risk of Bias tool, including random sequence generation, allocation concealment, blinding of participants and personnel, blinding of outcome assessment, incomplete outcome data, and selective reporting (Higgins et al., 2011).

### Statistical Analysis

Study heterogeneity was assessed through Q statistics and I-squared. We regarded I-squared <25%, 25-50%, and >50% as low, moderate, and high heterogeneity, respectively. The effect of NMBA on outcomes in ARDS patients was shown as relative risk (RR) with 95% confidence interval (CI). The random-effect model was used if I-squared >50%. In addition, publication bias was evaluated by funnel plots or Egger's linear regression test. All analyses were performed with STATA version 12.0 (Stata Corp., College Station, TX, USA). A *P* value <0.05 was considered as a statistically significant difference.

## Results

**Figure 1:**
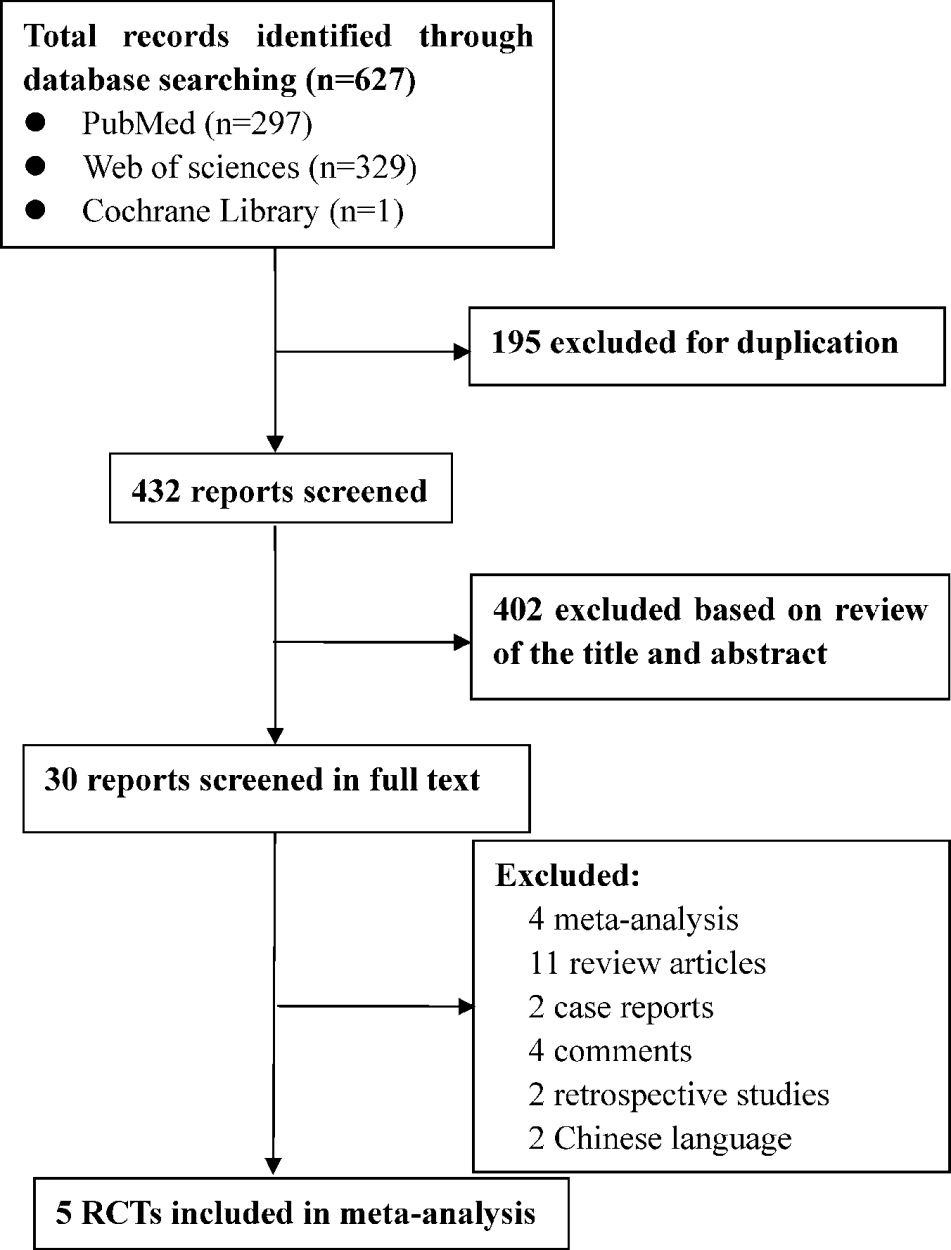
Flow chart of the study selection strategy.

A flow diagram of the screening strategy for inclusion in the meta-analysis is displayed in **Figure 1**. A total of 627 results were identified according to the search strategy: PubMed (*n* = 297), Web of Sciences (*n* = 329), and the Cochrane Library (*n* = 1). Among these, 195 were excluded because they were duplicates, and 402 were excluded because they did not meet the inclusion criteria after independently reviewing their titles and abstracts. The remaining 30 records were considered to be of relevance and the full papers were carefully screened. Four meta-analyses, 11 review articles, two case reports, four comments, two retrospective studies, and two studies in Chinese language were discarded. Finally, five eligible randomized controlled trials met the selection criteria (Papazian et al., 2010; Higgins et al., 2011; Alhazzani et al., 2013; Guervilly et al., 2017; Moss et al., 2019). A total of 1,461 ARDS patients were included, where 724 were treated by NMBA. The detailed characteristics of these studies are listed in **Tables 1** and **2**. ARDS was defined by a PaO_2_:FIO_2_ ratio <150 mmHg in four studies (Gainnier et al., 2004; Papazian et al., 2010; Guervilly et al., 2017; Moss et al., 2019) and in the remaining study by a PaO_2_:FIO_2_ ratio <200 mmHg (Forel et al., 2006). All the intervention groups received 48 h cisatracurium infusion for myorelaxation. In three randomized controlled trials, the sample size was less than 100 patients (Gainnier et al., 2004; Forel et al., 2006; Guervilly et al., 2017). The risk of bias assessment is shown in **Table 3**. A double-blind method with a low risk of bias (Papazian et al., 2010) was used in only one study.

**Table 1:** Study characteristics.

Study	Number of centers	Definition of ARDS	Sample size	Location	Sedation strategy	NMBA usage
Gainnier, 2004	4	PaO_2_:FIO_2_ ratio <150 mmHg and PEEP ≥5cmH_2_O	56	France	Midazolam and sufentanil	A bolus of 50 mg cisatracurium, followed by 5 μg/(kg∙min) infusion for 48 h.
Forel, 2006	3	PaO_2_:FIO_2_ ratio <200 mmHg and PEEP ≥5cmH_2_O	36	France	Midazolam and sufentanil	A bolus of 0.2 mg/kg cisatracurium, followed by 5 μg/(kg∙min) infusion for 48 h.
Papazian, 2010	20	PaO_2_:FIO_2_ ratio < 150 mmHg and PEEP ≥5cmH_2_O	339	France	Midazolam, sufentanil, Ketamin and propofol	A bolus of 15 mg cisatracurium followed by 37.5 mg per hour for 48 hours.
Guervilly, 2017	2	PaO_2_:FIO_2_ ratio <150 mmHg and PEEP ≥5cmH_2_O	24	France	Midazolam and sufentanil	A bolus of 15 mg cisatracurium, followed 37.5 mg per hour for 48 hours.
Moss, 2019	13	PaO_2_:FIO_2_ ratio <150 mmHg and PEEP ≥8cmH_2_O	1006	America	Not mandate	A bolus of 15 mg cisatracurium, followed 37.5 mg per hour for 48 hours.

NMBA, neuromuscular blocking agents; ARDS, acute respiratory distress syndrome; PEEP, positive end-expiratory pressure; ICU, intensive care unit.

**Table 2:** Characteristics of NMBA and control group in included studies.

Characteristics	Gainnier, 2004		Forel, 2006		Papazian, 2010		Guervilly, 2017		Moss, 2019	
	NMBA	Control	NMBA	Control	NMBA	Control	NMBA	Control	NMBA	Control
Number of patients	28	28	18	18	177	162	13	11	501	505
Age	59.8 ± 17.5	61.5 ± 14.6	52.0 ± 16.0	61.0 ± 18.0	58.0 ± 16.0	58 ± 15.0	72 (63–79)	60 (52–75)	56.6 ± 14.7	55.1 ± 15.9
Male sex	21 (75)	20 (71)	14 (78)	12 (67)	–	–	9 (69)	10 (91)	291 (58)	269 (53)
Sedation scale	Ramsay 6	Ramsay 6	Ramsay 6	Ramsay 6	Ramsay 6	Ramsay 6	Ramsay 6	Ramsay 6	Ramsay 6	Ramsay 2-3
SAPS II	41.8 ± 10.4	45.4 ± 10.5	47.0 ± 15.0	49.0 ± 19.0	50.0 ± 16.0	47.0 ± 14.0	47 (37–54)	48 (42–62)	–	–
PEEP, cmH2O	11.1 ± 2.8	10.9 ± 2.4	13.2 ± 2.7	11.0 ± 2.7	9.2 ± 3.2	9.2 ± 3.5	11 (10-11.5)	10 (9–12)	12.6 ± 3.6	12.5 ± 3.6
Plateau pressure, cmH_2_O	27.1 ± 6.2	26.1 ± 4.0	27.5 ± 4.4	24.8 ± 5.7	25.0 ± 5.1	24.4 ± 4.7	23 (19–26)	21 (19–25)	25.5 ± 6.0	25.7 ± 6.1
PaO_2_/FiO_2_	130 ± 34	119 ± 31	105 ± 22	125 ± 20	106.0± 36.0	115.0± 41.0	158 (131–185)	150 (121–187)	98.7 ± 27.9	99.5 ± 27.9
FiO_2_, %	70.2 ± 17.0	67.3 ± 15.8	80.0 ± 15.0	71.0 ± 19.0	79.0 ± 19.0	77.0 ± 22.0	60 (50–60)	50 (50–70)	80 ± 20	80 ± 20
PaCO_2_, mmHg	48.3 ± 9.0	47.4 ± 11.2	51.1 ± 9.9	47.2 ± 9.8	47.0 ± 11.0	47.0 ± 11.0	43(36-44)	43(37-52)	–	–
VT, mL/kg	7.1 ± 1.1	7.4 ± 1.9	6.5 ± 0.7	7.0 ± 0.7	6.55 ± 1.12	6.48 ± 0.92	6.2 (5.9-6.8)	6.3 (6.0-6.9)	6.3 ± 0.9	6.3 ± 0.9
Days free of ventilation at day 28	3.7 ± 7.2	1.7 ± 5.3	6.0 ± 8.6	5.4 ± 6.4	10.6 ± 9.7	8.5 ± 9.4	7 (0–20)	8 (0–18)	9.6 ± 10.4	9.9 ± 10.9
ICU mortality	13 (46.4)	20 (71.4)	5 (27.8)	10 (55.6)	52 (29.4)	63 (28.9)	5 (38)	3 (27)	–	–
28 day mortality	10 (35.7)	17 (60.7)	–	–	42 (23.7)	54 (33.3)	–	–	184 (36.7)	187 (37.0)
Barotrauma	0	1 (3.6)	0	0	9 (5.1)	19 (11.7)	–	–	20 (4.0)	32 (6.3)
ICU-acquired weakness, no./total no. (%)	0	0	1/18 (5.6)	1/18 (5.6)	40/112 (35.7)	28/89 (31.5)	–	–	107/226 (47.3)	89/228 (39)

NMBA, neuromuscular blocking agents; SAPS, simplified acute physiology score; PEEP, positive end-expiratory pressure; FiO_2,_ Fraction of inspired oxygen; VT, tidal volume; ICU, intensive care unit.

**Table 3:** Risk of bias assessment.

Study	Random sequence generation	Allocation concealment	Blinding of participants and personnel	Blinding of outcome assessment	Incomplete outcome data	Selective reporting	Anything else, ideally prespecified
Gainnier, 2004	Low risk of bias computer-generated	Low risk of bias Centralized	High risk of bias Nurses aware of assignment	High risk of bias Analyst aware of assignment	Low risk of bias outcome data complete	Low risk of bias None	Low risk of bias None
Forel, 2006	Low risk of bias computer-generated	Low risk of bias Centralized	High risk of bias Nurses aware of assignment	High risk of bias Evaluators aware of assignment	Low risk of bias outcome data complete	Low risk of bias None	Low risk of bias None
Papazian, 2010	Low risk of bias computer-generated	Low risk of bias Centralized	Low risk of bias Blinding of all participants	Low risk of bias Blinding of evaluators	Low risk of bias outcome data complete	Low risk of bias None	Low risk of bias None
Guervilly, 2017	Low risk of bias computer-generated	unclear risk of bias	unclear risk of bias	unclear risk of bias	Low risk of bias outcome data complete	Low risk of bias None	Low risk of bias None
Moss, 2019	Low risk of bias computer-generated	Low risk of bias Centralized	High risk of bias Participants aware of assignment	High risk of bias Evaluators aware of assignment	Low risk of bias outcome data complete	Low risk of bias None	Low risk of bias None

**Figure 2:**
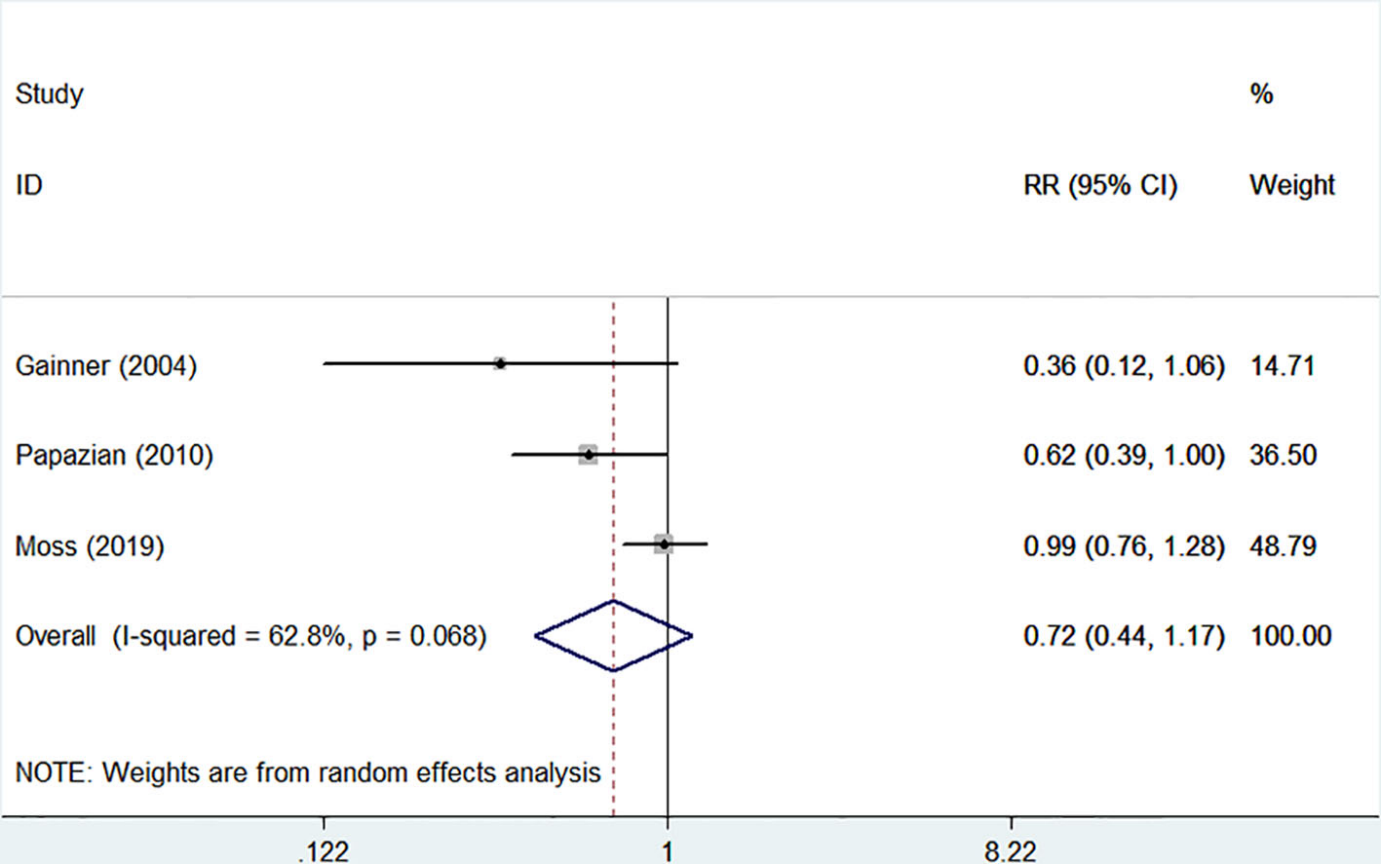
Forest plot of RR and 95% CI for 28-day mortality. Three studies included 1,401 patients (706 in the NMBA group vs. 695 in the control group). The overall estimate based on the random-effect model showed that NMBA infusion did not reduce the 28-day mortality (33.4% vs. 37.1%, RR = 0.72, 95% CI 0.44 to 1.17, *P* = 0.180, I-squared = 62.8%).

The 28-day mortality was reported in three studies (Gainnier et al., 2004; Papazian et al., 2010; Moss et al., 2019), including 1,401 patients (706 in the NMBA group vs. 695 in the control group). According to Egger's test, there were no significant differences for publication bias among these studies (*P* = 0.179), while the heterogeneity was statistically significant (I-squared = 62.8%, *P* = 0.068). The overall estimate based on the random-effect model showed that NMBA infusion did not reduce the 28-day mortality (33.4% vs. 37.1%, RR = 0.72, 95% CI 0.44 to 1.17, *P* = 0.180, **Figure 2**). Subgroup analysis without the ROSE trial showed that the use of NMBA decreased the 28-day mortality (RR = 0.57, 95% CI 0.37 to 0.88, *P* = 0.011, I-squared = 0).

**Figure 3:**
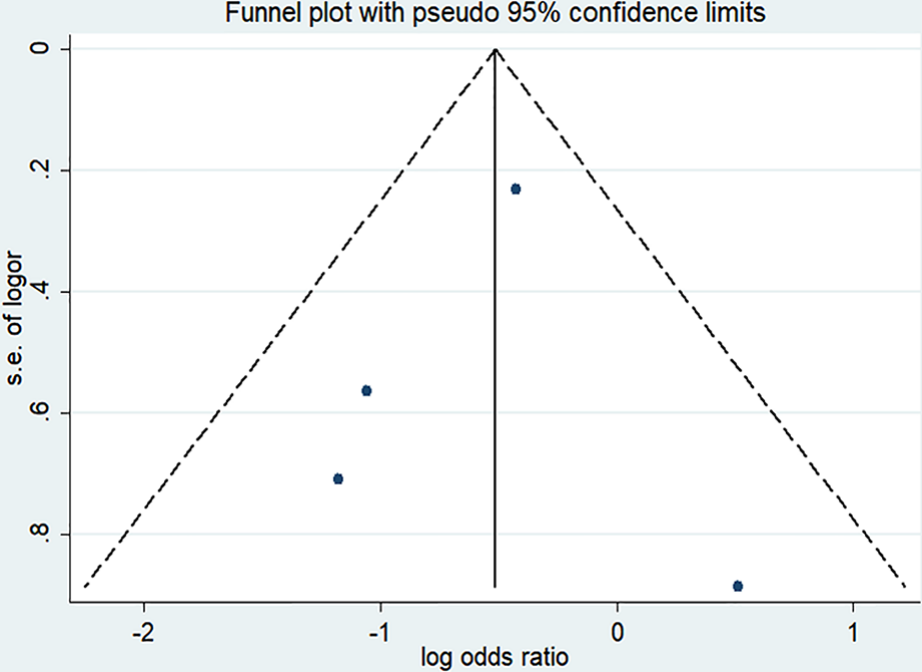
Funnel plot of studies discussing ICU mortality. The funnel plot did not show distinct asymmetry.

**Figure 4:**
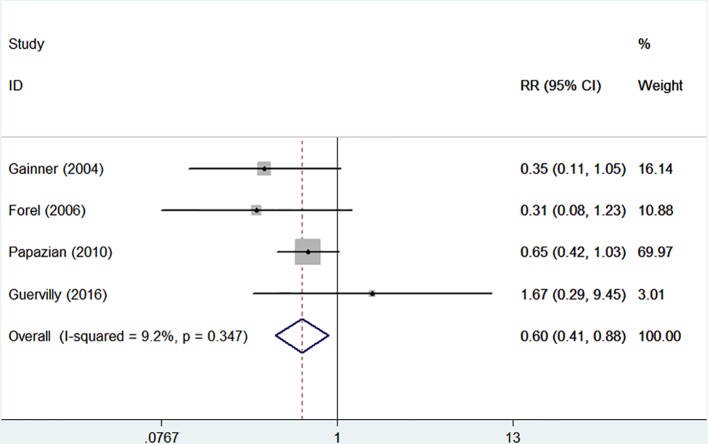
Forest plot of RR and 95% CI for ICU mortality. Four studies (235/455 patients treated by NMBA) that discussed the ICU mortality. The incidence of ICU mortality was 31.9% in the NMBA group and 43.6% in the control group. The pooled estimate showed that NMBA infusion was associated with lower ICU mortality based on the fixed-effect model (RR = 0.60, 95% CI 0.41 to 0.88, *P* = 0.009, I-squared = 9.2%).

There were four randomized controlled trials (235/455 patients treated by NMBA) that discussed the ICU mortality (Gainnier et al., 2004; Forel et al., 2006; Papazian et al., 2010; Guervilly et al., 2017). A funnel plot did not show distinct asymmetry (**Figure 3**). The Egger's linear regression test did not suggest publication bias (*P* = 0.815). The incidence of ICU mortality was 31.9% in the NMBA group and 43.6% in the control group. The pooled estimate showed that NMBA infusion was associated with lower ICU mortality based on the fixed-effect model (RR = 0.60, 95% CI 0.41 to 0.88, *P* = 0.009, I-squared = 9.2%, **Figure 4**).

**Figure 5:**
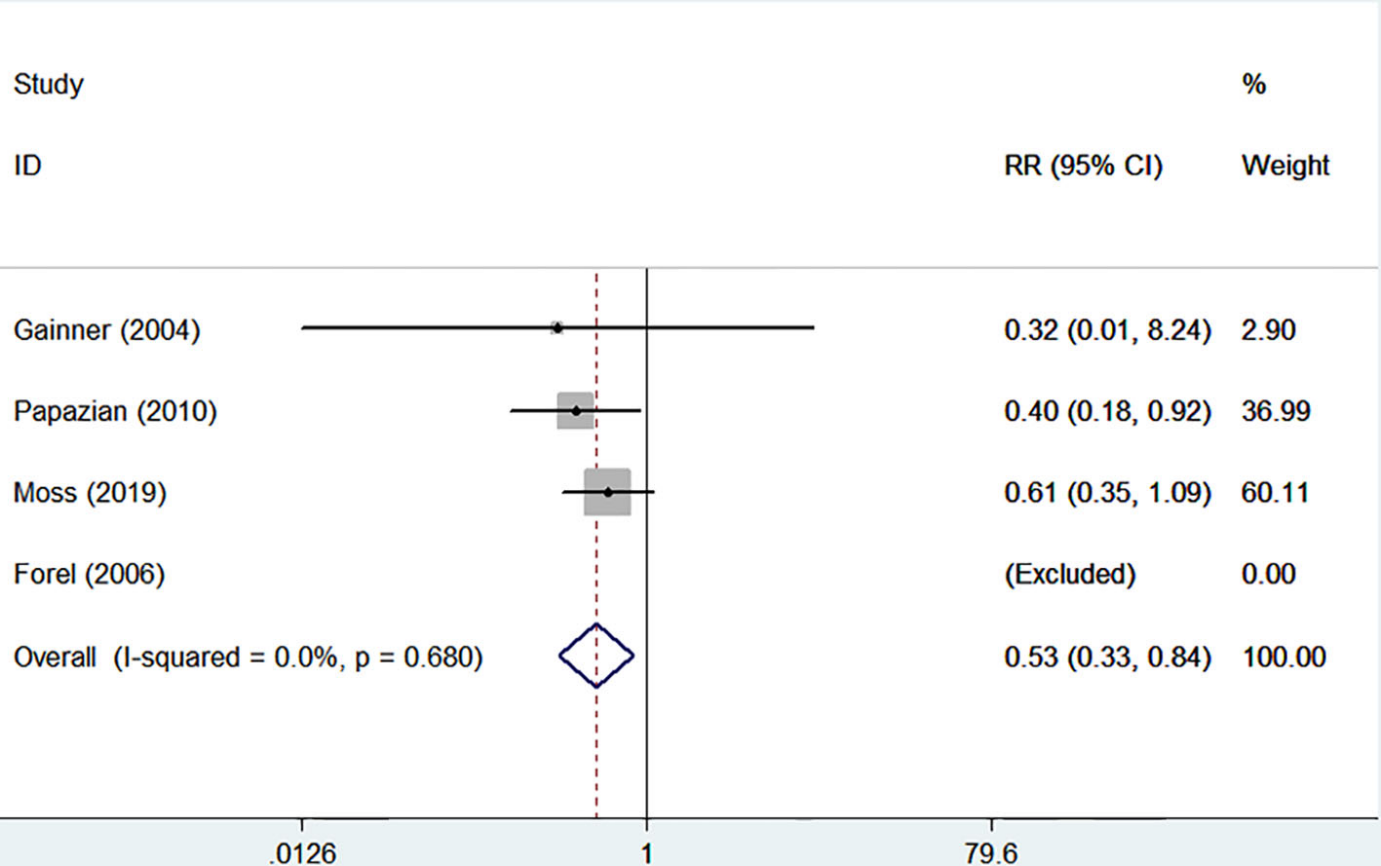
Forest plot of RR and 95% CI for the incidence of barotrauma. The incidence of barotrauma was described in four studies with 1,437 patients (724 in NMBA group vs. 713 in control group). The meta-regression results with fixed-effects analysis suggested that the incidence of barotrauma was significantly lower in patients treated with NMBA (RR = 0.53, 95% CI 0.33 to 0.84, *P* = 0.007, I-squared = 0).

**Figure 6:**
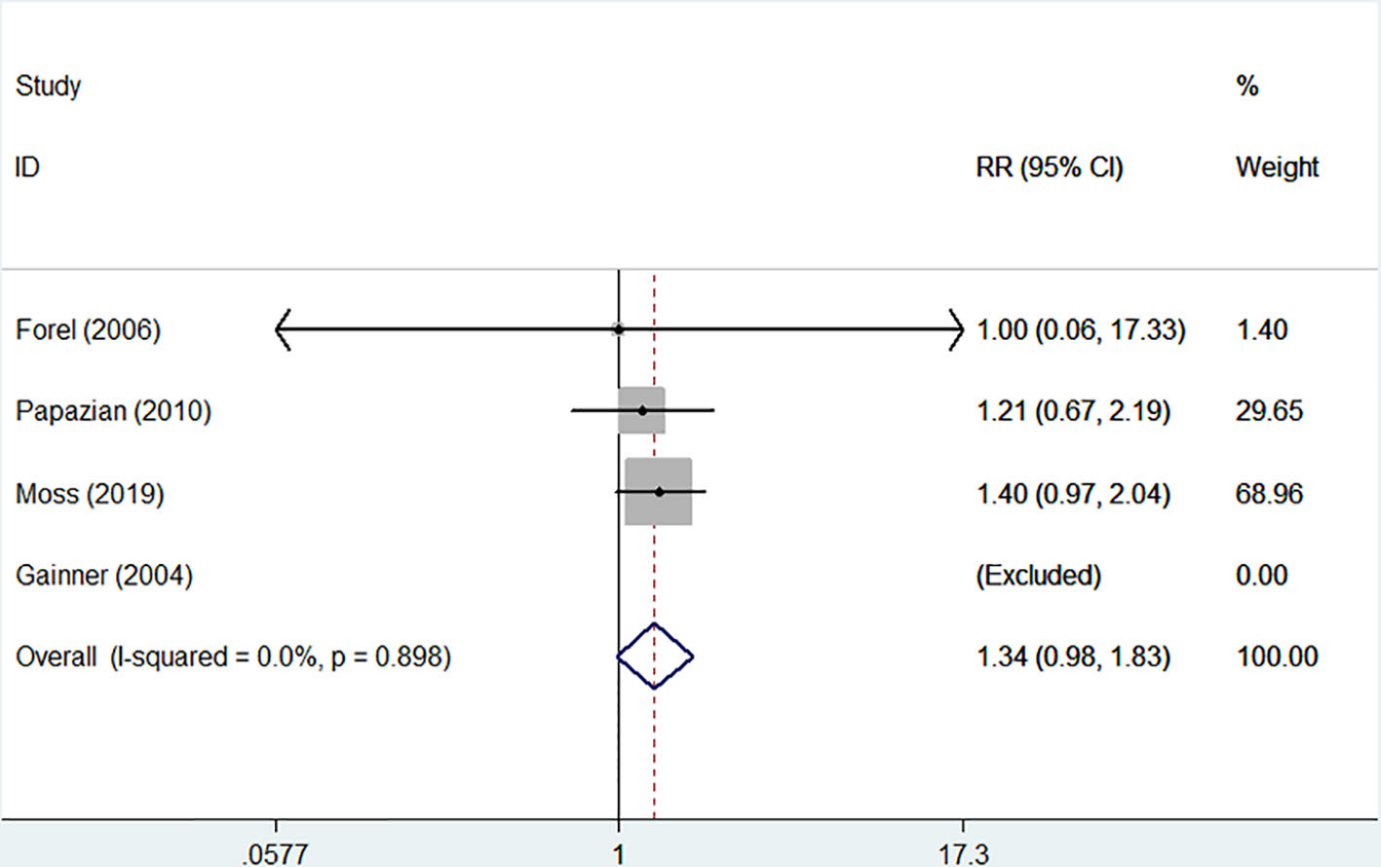
Forest plot of RR and 95% CI for the incidence of ICU-acquired weakness. The incidence of ICU-acquired weakness was described in four studies with 1,437 patients (724 in NMBA group vs. 713 in control group). Infusion of NMBA might increase the risk of ICU-acquired weakness (RR = 1.34, 95% CI 0.98 to 1.84, *P* = 0.066, I-squared = 0).

The incidences of barotrauma and ICU-acquired weakness were described in four randomized controlled trials with 1,437 patients (724 in NMBA group vs. 713 in control group) (Gainnier et al., 2004; Forel et al., 2006; Papazian et al., 2010; Moss et al., 2019). The publication bias was not significantly different (*P* = 0.770). A low degree of nonsignificant heterogeneity was present (I-squared = 0). The meta-regression results with fixed-effects analysis suggested that the incidence of barotrauma was significantly lower in patients treated with NMBA (RR = 0.53, 95% CI 0.33 to 0.84, *P* = 0.007, **Figure 5**). However, infusion of NMBA might increase the risk of ICU-acquired weakness based on the pooled estimate across the four studies (RR = 1.34, 95% CI 0.98 to 1.84, *P* = 0.066, I-squared = 0, **Figure 6**).

## Discussion

Early NMBA treatment did not reduce the 28-day mortality in patients with moderate-to-severe ARDS. This result was mainly based on the study with the largest sample size (Moss et al., 2019). However, we found that the ICU mortality and incidence of barotrauma were lower in patients treated with NMBA. However, NMBA infusion may increase the risk of ICU-acquired weakness.

ARDS is an inflammatory disease that can lead to alveolar injury. The hallmark of ARDS is refractory hypoxia due to ventilation-perfusion mismatch (Sweeney and McAuley, 2016). Mechanical ventilation is a conventional treatment for patients with ARDS (Fan et al., 2017). Patient-ventilator dyssynchrony, defined as a mismatch between the ventilatory needs of the patients and the amount of ventilation delivered, is a common problem present in about one-third of mechanically ventilated patients (Pham et al., 2017). In a period of low tidal volume ventilation in ARDS, the high metabolic rate and hypercarbia create significant ventilatory drive, leading to a greater risk of patient-ventilator dyssynchrony and a subsequent risk of volutrauma and barotrauma (Brochard et al., 2017; Papazian et al., 2019). NMBA has been suggested as a solution to this issue, because it can relax the skeletal muscles and achieve better patient-ventilatory synchrony and tidal volume control (Bourenne et al., 2017). Gainnier *et al*. were the first to evaluate the effects of NMBA infusion in ARDS patients. They conducted a randomized controlled trial of 56 ARDS patients with a PaO_2_/FIO_2_ ratio of <150 mmHg at a positive end-expiratory pressure (PEEP) of >5cm of water. The results revealed that NMBA usage could improve oxygenation and slightly reduce the ICU mortality (*P* = 0.057) (Gainnier et al., 2004). Furthermore, Forel and co-workers focused on another effect of NMBA in ARDS. They discovered that early NMBA administration decreases the proinflammatory response. However, no significant difference was found in ICU mortality due to the small sample size (*n* = 36) (Forel et al., 2006). A randomized controlled trial, the ARDS et Curarisation Systematique (ACURASYS) study, with a relatively large sample size (*n* = 339) was published in 2010 (Papazian et al., 2010). This study provided evidence to support the use of NMBA in ARDS patients. The ACURASYS study demonstrated an improvement in 90-day survival without increasing the risk of ICU-acquired weakness in patients with severe ARDS. The meta-analysis of these three randomized controlled trials confirmed the benefits of NMBA use in ARDS patients (Neto et al., 2012; Alhazzani et al., 2013). In contrast, the recently published ROSE trial did not demonstrate similar mortality reductions in what is the largest clinical study on this topic to date (Moss et al., 2019). Due to these inconsistencies, we conducted this updated meta-analysis to further evaluate the effect of NMBA in ARDS patients. Nonetheless, the impact of NMBA infusion on mortality mainly depended on the ROSE trial (Moss et al., 2019). In evaluating the ICU mortality without the ROSE trial, the effect of NMBA was favorable, whereas no significant difference was found when evaluating the 28-day mortality with this randomized controlled trial included. There are several reasons that might account for the inconformity between the ROSE trial and the ACURASYS study.

First, lung protective ventilation is an accepted and effective treatment for patients with ARDS, which decreases the short-term and long-term mortality owing to its reductions in inflammation and ventilator-induced lung injury (VILI) (Needham et al., 2012). The benefit of NMBA in the ACURASYS trial was only realized when the plateau pressure was incorporated into the multivariate analysis, suggesting that lung protective ventilation was perhaps less effectively applied in this trial compared to the ROSE trial. In addition, the ROSE trial enrolled patients much earlier than the ACURASYS trial. This raises the possibility that patients who experienced rapid improvement due to alveolar recruitment were included in the ROSE population, which potentially attenuated the NMBA signal.

Second, the mean PEEP in the ROSE trial was about 12 mmHg, which was higher than in the ACURASYS study (Papazian et al., 2010). A previous study has shown that spontaneous breathing causes occult pendelluft in ARDS, which over-inflates the dependent lung near the diaphragm, and this could be mitigated by high PEEP (Yoshida et al., 2013a; Yoshida et al., 2016; Morais et al., 2018). The higher mean level of PEEP in the ROSE trial could also have offered a greater degree of alveolar recruitment and attenuated the degree of hypoxemia and hypercapnia, which is one of the driving mechanisms of patient-ventilator dyssynchrony (Santa Cruz et al., 2013). Thus, one reason why the ROSE trial was negative, but the ACURASYS trial was positive, is that the low PEEP in the ACURASYS trial caused more VILI, which is why NMBA helped. However, in the ROSE trial, the high PEEP made NMBA unnecessary for reducing VILI.

Third, there was a substantially higher proportion of patients in the ACURASYS study who received prone positioning compared to less than 16% in the ROSE trial. In the ROSE trial, 20% of the patients were excluded for already using NMBA, whereas only 4% of patients in the ACURASYS study were excluded. These facts raise concern for a significant risk of selection bias, and attenuation of the treatment signal due to inclusion of a subset of patients with less physiologically severe lung injury. In addition, there was substantial cross-over between treatment groups in the ROSE trial, which may also lead to confounding. Finally, the patients in the ACURASYS trial were all deeply sedated, while only the patients in the intervention group were deeply sedated in the ROSE trial. Sedation is frequently prescribed in patients with ARDS, which facilitates tolerance of the intubation tube, reduces discomfort, and in some cases can improve patient-ventilator synchrony (Bourenne et al., 2017). Previous studies have indicated that early deep sedation can predispose a patient to double triggering and VILI, in addition to delirium, a longer time on mechanical ventilation or longer stay in the ICU, and an increased risk of ventilator-associated pneumonia (Balzer et al., 2015; Shah et al., 2017). However, minimizing sedation had beneficial effects in critically ill patients, improving the ability to participate in early exercise and rehabilitation in patients with ARDS (Kayambu et al., 2013). Early mobilization was associated with a shortened length of stay in the ICU and improved physical function, potentially aiding in recovery from ARDS (Schaller et al., 2016). The mismatch of sedation strategies between the two groups in the ROSE trial might contribute to the neutral effect of NMBA in ARDS. Therefore, we assumed that NMBA treatment could reduce the mortality according to our results.

We also found that the incidence of barotrauma was significantly lower in patients treated by NMBA (*P* = 0.007). Pulmonary barotrauma is a complication of mechanical ventilation, occurring in about 6.5% of ARDS patients and portending a poor outcome (Anzueto et al., 2004). The following aspects could account for the high incidence of barotrauma in ARDS patients. First, it is known that ARDS is a heterogeneous syndrome, in which atelectasis and edema are preferentially distributed to dependent lung regions, while independent lung regions are relatively well aerated (van der Zee and Gommers, 2019). The positive pressure ventilation may theoretically lead to overdistention of relatively normal alveoli and ultimately barotrauma. Second, the permissive hypercapnia strategy commonly employed in the setting of a state of high ventilatory demand is also a contributing risk factor for barotrauma (Papazian et al., 2019). Third, spontaneous breathing is involved in the barotrauma. In experimental studies, spontaneous breathing was associated with high transpulmonary pressure, which worsened injury in animals with severe lung injury (Yoshida et al., 2012; Yoshida et al., 2013b). The vigorous spontaneous effort increased the risk of the dyssynchrony between the patients' spontaneous effort and the ventilator, which can worsen lung injury (Yoshida et al., 2017). NMBA infusion could abolish spontaneous ventilatory activity and safeguard smooth implementation of lung protective ventilation, subsequently decreasing the occurrence of barotrauma.

Even so, the increased risk of ICU-acquired weakness needs attention. In our meta-analysis, we found that the incidence of ICU-acquired weakness was higher in patients who received NMBA treatment. This was in accordance with previous studies. Use of NMBA was an independent risk factor for ICU-acquired weakness, which subsequently increased the duration of mechanical ventilation and mortality (Jolley et al., 2016). The potential mechanism was unclear. Recent data have shown that NMBA may not be associated with ICU-acquired weakness when used for less than 48 h (Kress and Hall, 2014). This conclusion remains inconsistent; thus, it needs to be further investigated. Although, some interventions could be considered to reduce the incidence of ICU-acquired weakness in patients receiving NMBA treatment, such as intensive insulin therapy, neuromuscular stimulation, and early exercise (Zorowitz, 2016). However, the high risk of life-threatening hypoglycemia limits the application of intensive insulin therapy in clinical practice (Hermans et al., 2014). Early mobilization may be an effective measure to reduce ICU-acquired weakness (Zhang et al., 2019).

According to our analysis, we believe that the use of NMBA is still important for severe ARDS patients with refractory hypoxemia and patient-ventilator dyssynchrony, and at high risk of early barotrauma. However, it cannot be overused in patients with less severe ARDS, rapid improvement with initial ventilator strategies, and adequate lung protection and ventilator synchrony with light sedation, especially considering the signal towards an increased ICU-acquired weakness in NMBA patients and the signal suggesting an increased risk of cardiovascular events in the ROSE trial (Moss et al., 2019).

## Limitations

There were some limitations in our study that should be pointed out. First, we did not evaluate the effect of other NMBA, except for cisatracurium, in ARDS, because all the studies included used cisatracurium infusion in the treatment group. Second, the dose-dependent effects of NMBA in ARDS were not fully understood due to the restrictions of the meta-analysis. Third, the inherent weaknesses of meta-analysis, such as publication bias and data combined from different protocols, might impact the results. Finally, the ICU mortality was not analyzed in recently published research (Alhazzani et al., 2013). The beneficial effect of NMBA on ICU mortality might diminish with the inclusion of this study. However, there were some inconsistencies between experimental and control groups in the published study. The real effects of NMBA need to be further evaluated and confirmed by a study with a stricter design.

## Conclusion

The results of the meta-analysis presented here support the use of NMBA in patients with moderate-to-severe ARDS. NMBA could reduce ICU mortality and the incidence of barotrauma. However, a continuous NMBA infusion needs to be used with caution, because it may increase the risk of ICU-acquired weakness. On account of the limitations in the ROSE trial, the effect of NMBA on mortality in ARDS patients needs to be clarified by further high-quality randomized controlled trials. Meanwhile, future studies should focus on the incidence of barotrauma, which may be a more appropriate surrogate outcome to examine in the study of the beneficial effects of NMBA, and this variable strongly favored this intervention. In addition, a greater awareness is needed of the increased risk of ICU-acquired weakness in patients undergoing NMBA treatment. Further research on ICU-acquired weakness is needed to better understand its pathophysiology, so that more effective interventions may be developed.

## Author Contributions

T-hQ and S-hW conceived and designed the study. X-lL and W-xG performed abstract screening and data extraction. X-bW and Z-hW analyzed the data and drafted the manuscript. All authors revised and approved the final manuscript.

## Funding

This study was supported by grants from Medical Science and Technology Research Funding of Guangdong (grant no. A2019409), the Fundamental Research Funds for the Central Universities (grant no. 2019MS136), Science and Technology Projects of Guangzhou (grant no. 201903010097) and National Clinical Key Specialty Construction Project of China (grant nos. 2012-649 and 2013-544). The funders had no role in the study design, data collection and analysis, decision to publish, nor preparation of the manuscript. The work was not funded by any industry sponsors. All authors agreed to submit the manuscript for publication.

## Conflict of Interest

The authors declare that the research was conducted in the absence of any commercial or financial relationships that could be construed as a potential conflict of interest.

## Abbreviations

ACURASYS, ARDS et Curarisation Systematique; ARDS, acute respiratory distress syndrome; CI, confidence interval; ICU, intensive care unit; NMBA, neuromuscular blocking agents; PEEP: positive end-expiratory pressure; ROSE, Reevaluation of Systemic Early Neuromuscular Blockade; RR, relative risk.

## References

[B1] Alhazzani W. Alshahrani M. Jaeschke R. Forel J. M. Papazian L. Sevransky J. (2013). Neuromuscular blocking agents in acute respiratory distress syndrome: a systematic review and meta-analysis of randomized controlled trials. Crit. Care 17, R43. 10.1186/cc12557 23497608 PMC3672502

[B2] Anzueto A. Frutos-Vivar F. Esteban A. Alía I. Brochard L. Stewart T. (2004). Incidence, risk factors and outcome of barotrauma in mechanically ventilated patients. Intensive Care Med. 30, 612–619. 10.1007/s00134-004-2187-7 14991090

[B3] Balzer F. Weiß B. Kumpf O. Treskatsch S. Spies C. Wernecke K. D. (2015). Early deep sedation is associated with decreased in-hospital and two-year follow-up survival. Crit. Care 19, 197. 10.1186/s13054-015-0929-2 25928417 PMC4435917

[B4] Bellani G. Laffey J. G. Pham T. Fan E. Brochard L. Esteban A. (2016). Epidemiology, patterns of care, and mortality for patients with acute respiratory distress syndrome in intensive care units in 50 countries. JAMA 315, 788–800. 10.1001/jama.2016.0291 26903337

[B5] Blanch L. Villagra A. Sales B. Montanya J. Lucangelo U. Luján M. (2015). Asynchronies during mechanical ventilation are associated with mortality. Intensive Care Med. 41, 633–641. 10.1007/s00134-015-3692-6 25693449

[B6] Bourenne J. Hraiech S. Roch A. Gainnier M. Papazian L. Forel J. M. (2017). Sedation and neuromuscular blocking agents in acute respiratory distress syndrome. Ann. Transl. Med. 5, 291. 10.21037/atm.2017.07.19 28828366 PMC5537113

[B7] Brochard L. Slutsky A. Pesenti A. (2017). Mechanical ventilation to minimize progression of lung injury in acute respiratory failure. Am. J. Respir. Crit. Care Med. 195, 438–442. 10.1164/rccm.201605-1081CP 27626833

[B8] Fan E. Del S. L. Goligher E. C. Hodgson C. L. Munshi L. Walkey A. J. (2017). An official American Thoracic Society/European society of intensive care Medicine/Society of critical care medicine clinical practice guideline: mechanical ventilation in adult patients with acute respiratory distress syndrome. Am. J. Respir. Crit. Care Med. 195, 1253–1263. 10.1164/rccm.201703-0548ST 28459336

[B9] Fan E. Brodie D. Slutsky A. S. (2018). Acute respiratory distress syndrome: advances in diagnosis and treatment. JAMA 319, 698–710. 10.1001/jama.2017.21907 29466596

[B10] Forel J. M. Roch A. Marin V. Michelet P. Demory D. Blache J. L. (2006). Neuromuscular blocking agents decrease inflammatory response in patients presenting with acute respiratory distress syndrome. Crit. Care Med. 34, 2749–2757. 10.1097/01.CCM.0000239435.87433.0D 16932229

[B11] Gainnier M. Roch A. Forel J. M. Thirion X. Arnal J. M. Donati S. (2004). Effect of neuromuscular blocking agents on gas exchange in patients presenting with acute respiratory distress syndrome. Crit. Care Med. 32, 113–119. 10.1097/01.CCM.0000104114.72614.BC 14707568

[B12] Guervilly C. Bisbal M. Forel J. M. Mechati M. Lehingue S. Bourenne J. (2017). Effects of neuromuscular blockers on transpulmonary pressures in moderate to severe acute respiratory distress syndrome. Intensive Care Med. 43, 408–418. 10.1007/s00134-016-4653-4 28013329

[B13] Hermans G. De Jonghe B. Bruyninckx F. Van den Berghe G. (2014). Interventions for preventing critical illness polyneuropathy and critical illness myopathy. Cochrane Database Syst Rev CD006832. 10.1002/14651858.CD006832.pub3 24477672 PMC7390458

[B14] Higgins J. P. Altman D. G. Gotzsche P. C. Juni P. Moher D. Oxman A. D. (2011). The cochrane collaboration's tool for assessing risk of bias in randomised trials. BMJ 343, d5928. 10.1136/bmj.d5928 22008217 PMC3196245

[B15] Jolley S. E. Bunnell A. E. Hough C. L. (2016). ICU-acquired weakness. Chest 150, 1129–1140. 10.1016/j.chest.2016.03.045 27063347 PMC5103015

[B16] Kayambu G. Boots R. Paratz J. (2013). Physical therapy for the critically ill in the ICU: a systematic review and meta-analysis. Crit. Care Med. 4, 1543–1554. 10.1097/CCM.0b013e31827ca637 23528802

[B17] Kress J. P. Hall J. B. (2014). ICU-acquired weakness and recovery from critical illness. N Engl. J. Med. 370, 1626–1635. 10.1056/NEJMra1209390 24758618

[B18] Morais C. C. A. Koyama Y. Yoshida T. Plens G. M. Gomes S. Lima C. A. S. (2018). High positive end-expiratory pressure renders spontaneous effort noninjurious. Am. J. Respir. Crit. Care Med. 197, 1285–1296. 10.1164/rccm.201706-1244OC 29323536 PMC5955057

[B19] Moss M. Huang D. T. Brower R. G. Ferguson N. D. Ginde A. A. Gong M. N. (2019). Early neuromuscular blockade in the acute respiratory distress syndrome. N Engl. J. Med. 380, 1997–2008. 10.1056/NEJMoa1901686 31112383 PMC6741345

[B20] Murray M. J. DeBlock H. Erstad B. Gray A. Jacobi J. Jordan C. (2016). Clinical practice guidelines for sustained neuromuscular blockade in the adult critically ill patient. Crit. Care Med. 44, 2079–2103. 10.1097/CCM.0000000000002027 27755068

[B21] Needham D. M. Colantuoni E. Mendez-Tellez P. A. Dinglas V. D. Sevransky J. E. Dennison Himmelfarb C. R. (2012). Lung protective mechanical ventilation and two year survival in patients with acute lung injury: prospective cohort study. BMJ 344, e2124. 10.1136/bmj.e2124 22491953 PMC3320566

[B22] Neto A. S. Pereira V. G. Esposito D. C. Damasceno M. C. Schultz M. J. (2012). Neuromuscular blocking agents in patients with acute respiratory distress syndrome: a summary of the current evidence from three randomized controlled trials. Ann. Intensive Care 2, 33. 10.1186/2110-5820-2-33 22835162 PMC3475105

[B23] Papazian L. Forel J. M. Gacouin A. Penot-Ragon C. Perrin G. Loundou A. (2010). Neuromuscular blockers in early acute respiratory distress syndrome. N Engl. J. Med. 363, 1107–1116. 10.1056/NEJMoa1005372 20843245

[B24] Papazian L. Aubron C. Brochard L. Chiche J. D. Combes A. Dreyfuss D. (2019). Formal guidelines: management of acute respiratory distress syndrome. Ann. Intensive Care 9, 69. 10.1186/s13613-019-0540-9 31197492 PMC6565761

[B25] Pham T. Brochard L. J. Slutsky A. S. (2017). Mechanical ventilation: state of the art. Mayo Clin. Proc. 92, 1382–1400. 10.1016/j.mayocp.2017.05.004 28870355

[B26] Santa Cruz R. Rojas J. I. Nervi R. Heredia R. Ciapponi A. (2013). High versus low positive end-expiratory pressure (PEEP) levels for mechanically ventilated adult patients with acute lung injury and acute respiratory distress syndrome. Cochrane Database Syst. Rev. CD009098. 10.1002/14651858.CD009098.pub2 23740697 PMC6517097

[B27] Schaller S. J. Anstey M. Blobner M. Edrich T. Grabitz S. D. Gradwohl-Matis I. (2016). Early, goal-directed mobilisation in the surgical intensive care unit: a randomised controlled trial. Lancet 388, 1377–1388. 10.1016/S0140-6736(16)31637-3 27707496

[B28] Shah F. A. Girard T. D. Yende S. (2017). Limiting sedation for patients with acute respiratory distress syndrome - time to wake up. Curr. Opin. Crit. Care 23, 45–51. 10.1097/MCC.0000000000000382 27898439 PMC5729753

[B29] Sweeney R. M. McAuley D. F. (2016). Acute respiratory distress syndrome. Lancet 388, 2416–2430. 10.1016/S0140-6736(16)00578-X 27133972 PMC7138018

[B30] Tao W. Yang L. Q. Gao J. Shao J. (2018). Neuromuscular blocking agents for adult patients with acute respiratory distress syndrome: a meta-analysis of randomized controlled trials. J. Trauma Acute Care Surg. 85, 1102–1109. 10.1097/TA.0000000000002057 30462621

[B31] van der Zee P. Gommers D. (2019). Recruitment maneuvers and higher PEEP, the so-called open lung concept, in Patients with ARDS. Crit. Care 23, 73. 1186/s13054-019-2365-1 30850004 10.1186/s13054-019-2365-1 PMC6408810

[B32] Yoshida T. Uchiyama A. Matsuura N. Mashimo T. Fujino Y. (2012). Spontaneous breathing during lung-protective ventilation in an experimental acute lung injury model: high transpulmonary pressure associated with strong spontaneous breathing effort may worsen lung injury. Crit. Care Med. 40, 1578–1585. 10.1097/CCM.0b013e3182451c40 22430241

[B33] Yoshida T. Torsani V. Gomes S. De Santis R. R. Beraldo M. A. Costa E. L. (2013a). Spontaneous effort causes occult pendelluft during mechanical ventilation. Am. J. Respir. Crit. Care Med. 188, 1420–1427. 10.1164/rccm.201303-0539OC 24199628

[B34] Yoshida T. Uchiyama A. Matsuura N. Mashimo T. Fujino Y. (2013b). The comparison of spontaneous breathing and muscle paralysis in two different severities of experimental lung injury. Crit. Care Med. 41, 536–545. 10.1097/CCM.0b013e3182711972 23263584

[B35] Yoshida T. Roldan R. Beraldo M. A. Torsani V. Gomes S. De Santis R. R. (2016). Spontaneous effort during mechanical ventilation: maximal injury with less positive end-expiratory pressure. Crit. Care Med. 44, e678–e688. 10.1097/CCM.0000000000001649 27002273

[B36] Yoshida T. Fujino Y. Amato M. B. Kavanagh B. P. (2017). Fifty years of research in ARDS. spontaneous breathing during mechanical ventilation. risks, mechanisms, and management. Am. J. Respir. Crit. Care Med. 195, 985–992. 10.1164/rccm.201604-0748CP 27786562

[B37] Zhang L. Hu W. Cai Z. Liu J. Wu J. Deng Y. (2019). Early mobilization of critically ill patients in the intensive care unit: a systematic review and meta-analysis. PloS One 14, e0223185. 10.1371/journal.pone.0223185 31581205 PMC6776357

[B38] Zorowitz R. D. (2016). ICU-Acquired weakness: a rehabilitation perspective of diagnosis, treatment, and functional management. Chest 150, 966–971. 10.1016/j.chest.2016.06.006 27312737

